# Pathophysiological Mechanisms of *Staphylococcus* Non-*aureus* Bone and Joint Infection: Interspecies Homogeneity and Specific Behavior of *S. pseudintermedius*

**DOI:** 10.3389/fmicb.2016.01063

**Published:** 2016-07-12

**Authors:** Yousef Maali, Patrícia Martins-Simões, Florent Valour, Daniel Bouvard, Jean-Philippe Rasigade, Michele Bes, Marisa Haenni, Tristan Ferry, Frédéric Laurent, Sophie Trouillet-Assant

**Affiliations:** ^1^Centre International de Recherche en Infectiologie, INSERM U1111, CNRS UMR5308, Université de Lyon 1, ENS de Lyon, Team “Pathogenesis of staphylococcal infections”Lyon, France; ^2^Department of Clinical Microbiology, Northern Hospital Group, Hospices Civils de LyonLyon, France; ^3^Infectious Diseases Department, Northern Hospital Group, Hospices Civils de LyonLyon, France; ^4^INSERM U1209, Albert Bonniot InstituteGrenoble, France; ^5^Université Grenoble AlpesGrenoble, France; ^6^National Reference Center of Staphylococci, Hospices Civils de LyonLyon, France; ^7^ANSES – French Agency for Food, Environmental and Occupational Health & SafetyLyon, France

**Keywords:** *Staphylococcus* non-*aureus*, *Staphylococcus pseudintermedius*, bone and joint infection (BJI), invasion, fibronectin, integrin α_5_β_1_

## Abstract

Implicated in more than 60% of bone and joint infections (BJIs), *Staphylococci* have a particular tropism for osteoarticular tissue and lead to difficult-to-treat clinical infections. To date, *Staphylococcus aureus* internalization in non-professional phagocytic cells (NPPCs) is a well-explored virulence mechanism involved in BJI chronicity. Conversely, the pathophysiological pathways associated with *Staphylococcus* non-*aureus* (SNA) BJIs have scarcely been studied despite their high prevalence. In this study, 15 reference strains from 15 different SNA species were compared in terms of (i) adhesion to human fibronectin based on adhesion microplate assays and (ii) internalization ability, intracellular persistence and cytotoxicity based on an *in vitro* infection model using human osteoblasts. Compared to *S. aureus*, *S. pseudintermedius* was the only species that significantly adhered to human fibronectin. This species was also associated with high (even superior to *S. aureus*) internalization ability, intracellular persistence and cytotoxicity. These findings were confirmed using a panel of 17 different *S. pseudintermedius* isolates. Additionally, *S. pseudintermedius* internalization by osteoblasts was completely abolished in β_1_ integrin-deficient murine osteoblasts. These results suggest the involvement of β_1_ integrin in the invasion process, although this mechanism was previously restricted to *S. aureus*. In summary, our results suggest that internalization into NPPCs is not a classical pathophysiologic mechanism of SNA BJIs. *S. pseudintermedius* appears to be an exception, and its ability to invade and subsequently induce cytotoxicity in NPPCs could explain its severe and necrotic forms of infection, notably in dogs, which exhibit a high prevalence of *S. pseudintermedius* infection.

## Introduction

Bone and joint infections (BJIs) represent a major public health issue, with an annual mortality rate near 5% and a classically reported relapse rate of 20–30% that can reach 80% in specific populations (Lentino, 2003). However, *Staphylococci* represent the most prevalent aetiologic agents, causing more than 60% of all BJIs. These pathogens are classically separated into two groups: (i) *Staphylococcus aureus*, involved in acute and chronic BJIs due to its large panel of virulence and persistence factors and (ii) *Staphylococcus* non-*aureus* (SNA) species, classically considered as less virulent, opportunistic pathogens that are primarily responsible for chronic implant-associated BJIs.

Several studies have explored the pathophysiology of *S. aureus* in BJIs and have elucidated mechanisms implicated in the course of chronic infections using *in vitro* and *in vivo* approaches. Two key mechanisms have been identified to explain the high rate of treatment failure of *S. aureus* BJIs: (i) the formation of biofilm, which is defined as the community of microbes that adhere to a biotic or an abiotic surface and that are covered by an adhesive protective matrix, and (ii) internalization and sanctuarization by which invasive bacteria actively induce their own uptake by phagocytosis into host cells, where they establish a protected niche within which they can replicate. Biofilm and intracellular lifestyles help *S. aureus* evade most antibiotics and the immune system. SNA is highly prevalent (even superior to *S. aureus* in some series) in cases of prosthetic joint infections (PJIs), of which *S. epidermidis* accounts for 30–43%. However, little is known regarding the behavior of the SNA species during the course of an infection (Zimmerli et al., 2004; Lourtet-Hascoett et al., 2015). Concerning biofilm formation, the few studies that have included panels of clinical isolates (i.e., *S. epidermidis*, *S. hominis*, *S. lugdunensis*) have highlighted a marked variability within each species (Seifert et al., 2005; Fredheim et al., 2009; Oliveira et al., 2015; Szczuka et al., 2015). Regarding the ability of SNA to be internalized by osteoblasts, the scarce data available are controversial. Indeed, using a single clinical strain isolated from BJI, Khalil et al. (2007) reported that *S. epidermidis* is able to invade human osteoblasts via a fibronectin-independent pathway. In contrast, we previously demonstrated using a large panel of clinical isolates that the extent of invasion in an *in vitro* infection model were so low that this mechanism is likely not involved in BJI with *S. epidermidis* (Valour et al., 2013). In the same way, Hussain et al. (2015) suggested that the *S. lugdunensis* invasion of endothelial, epithelial and fibroblastic cells is mediated by the bacterial autolysin AtlL whereas Campoccia et al. (2015) reported no internalization of this pathogen by human osteoblasts. In addition to these controversies, this issue remains under debate for all other *Staphylococci* species.

In this context, the aim of the present study was to determine whether the various SNA species exhibit distinct behaviors in terms of their capacities to adhere, invade and persist in osteoblasts under the same experimental conditions using an *in vitro* infection model. For this purpose, a screening approach using a panel of 15 reference isolates belonging to 15 different SNA species was employed. The original data obtained for the *S. pseudintermedius* reference strain led us to extend the investigation to include a set of 17 clinical isolates of this species and to explore the bacterial and cellular factors involved in the process of internalization using murine osteoblasts defective for β_1_ integrins.

## Materials and Methods

### Human Ethics Statement

This study was approved by the French South-East ethics committee (reference number 2013-018). In accordance with French legislation, written informed patient consent was not required for the use of the collected clinical isolates.

### Bacterial Strains and Culture Media

A collection of 15 reference strains of different SNA species was used (**Table 1**). The laboratory strain *S. aureus* 8325-4, well characterized for its ability to bind to fibronectin and to invade osteoblasts, was used as a positive control in each experiment. Its isogenic mutant, DU5883 (inactivated for the *fnbA/B* genes and therefore unable to adhere or to invade osteoblasts), was used as a negative control. Seventeen human and animal clinical *S. pseudintermedius* isolates were also included. The identities of all strains were confirmed via matrix-assisted laser desorption ionization time-of-flight mass spectrometry (MALDI-TOF-MS, bioMérieux, Marcy-L’Etoile, France). Details on the characteristics of the isolates are summarized in **Table 1**. Prior to the assays, strains were cultured overnight in brain heart infusion medium (BHI; bioMérieux) aerobically at 36°C.

**Table 1:** Description of the strains used in this study.

*Staphylococcus* spp. (Reference strains)			*S. pseudintermedius* (Clinical isolates)	
	
Species/sub-species	Strain	Origin	Isolate	Origin
*S. aureus*	8325-4	Human corneal ulcer	N°1	Human skin lesion
*S. aureus Δfnb*	DU 5883	Human corneal ulcer	N°2	Human nasal carriage
*S. intermedius*	CCM 5739	Pigeon nose	N°3	Human blood culture
*S. pseudintermedius*	LMG 22219	Cat lung tissue	N°4	Human CSF (Cerebrospinal fluid)
*S. capitis subsp. capitis*	CCM 2734	Human skin	N°5	Human skin lesion
*S. caprae*	CCM3573	Goat milk	N°6	Human pacemaker
*S. epidermidis*	CCM 2124	Human nose	N°7	Human bone biopsy
*S. gallinarum*	CCM 3572	Chicken skin	N°8	Human aspiration
*S. haemolyticus*	CCM 2737	Human skin	N°9	Human blood culture
*S. hominis subsp. hominis*	DSM 20328	Human skin	N°10	Human drain fluid
*S. lugdunensis*	ATCC 43809	Human axillary node	N°11	Dog skin pustule
*S. pettenkoferi*	CIP 107711	Human blood	N°12	Human hip prosthesis
*S. saprophyticus subsp.saprophyticus*	CCM 883	Human urine	N°13	Human leg abscess
*S. sciuri subsp. sciuri*	ATCC 29062	Gray squirrel skin	N°14	Human nasal carriage
*S. simulans*	ATCC 27848	Human skin	N°15	Human skin lesion
*S. warneri*	CCM 2730	Human Skin	N°16	Horse skin lesion
*S. xylosus*	ATCC 29971	Human skin	N°17	Human bone fistula


### Microplate Assay of Bacterial Adhesion to Fibronectin

The assays of bacterial adhesion to fibronectin were performed *in vitro* in 96-wells flat–bottom microplates, as described elsewhere (Rasigade et al., 2011). Briefly, the wells were coated with 200 μL of human fibronectin (Dutsher SAS, Brumath, France) at 50 μg/mL (18 h, 4°C). They were then washed three times (20 min, 37°C) with Phosphate-buffered saline (PBS) supplemented with 1% foetal bovine serum (FBS). Meanwhile, after an overnight broth culture in BHI medium, bacterial suspensions were adjusted to an OD_600_ of 1 ± 0.05, corresponding to a concentration of approximately 1 × 10^9^ cells/mL. Bacteria were then centrifuged (12,000 g, 2 min), and the pellets were washed and suspended in PBS. One hundred microliters of each bacterial suspension were incubated in the fibronectin-coated plates for 45 min at 37°C with mild shaking. The wells were then washed 3 times with PBS to remove non-adherent bacteria. Then, adherent bacteria were fixed with glutaraldehyde (2.5% v/v in 0.1 M PBS, 2 h at 4°C) and stained with crystal violet (0.1% m/v) for 30 min at room temperature. After 3 washes with PBS, the total remaining stain impregnating the adherent bacteria was solubilized by incubating the wells with Triton X-100 solution (0.2% v/v – H_2_O; Sigma–Aldrich, St. Louis, MO, USA) at room temperature for 30 min. The adhesion of bacteria to fibronectin was finally assessed spectrophotometrically by measuring the OD_620_ of each well using a plate reader (Auto Reader Model 680, Bio-Rad, Hercules, CA, USA). The results are expressed as the means ± standard deviation of the OD_620_ for 3 experiments performed in quadruplicate. The values obtained for each strain were normalized to those of the reference strain *S. aureus* 8325-4.

### Osteoblast Culture

All cell culture reagents were obtained from GIBCO (Paisley, United Kingdom). Human MG63 osteoblastic cells (LGC Standards, Teddington, UK) were cultured in Dulbecco’s modified Eagle’s medium (DMEM) containing 2 mM L-glutamine and 25 mM HEPES and supplemented with 10% FBS and 100 U/ml penicillin and streptomycin (“growth medium with antibiotics”). In addition, two murine osteoblastic cell lines were specifically used for the exploration of the internalization process: (i) the OB-β_1_^fl/fl^ cell line, expressing the functional integrin β_1_ subunit, (ii) the OB-β_1_^-/-^ cell line, deficient in the expression of the β_1_ integrin subunit after the conditional deletion of the *itgb1* gene. Both cell lines were derived from calvaria of transgenic mice bearing *itgb1-*floxed alleles. OB-β_1_^fl/fl^ cells were then immortalized as previously described via retroviral infection with the SV40 large T-antigen (Brunner et al., 2011). Next, OB-β_1_^-/-^ cells were obtained from the parental OB-β_1_^fl/fl^ cell line after expressing Cre recombinase. All cells were maintained at 1 passage per week.

### Determination of the Invasion Capacity and Persistence of *Staphylococcus* spp. in Osteoblasts

Osteoblasts were seeded at 80,000 cells per well on 24-well tissue culture plates (Falcon, Le Pont de Claix, France) in 1 mL of growth medium with antibiotics. One day later, the cells were washed twice with 1 mL of DMEM before the addition of bacteria. Bacterial suspensions in growth medium without antibiotics were added to the cell culture wells at a multiplicity of infection (MOI) of 100:1, as previously described (Trouillet et al., 2011). After 2 h of co-culture of the bacteria and osteoblasts in a 37°C/5% CO_2_ incubator, the wells were washed twice with 1 mL of DMEM. They were then incubated for 1 h in medium containing 200 μg/mL gentamicin to kill only the extracellular bacteria. The cells used for the measurement of intracellular persistence were then incubated in medium containing 40 μg/mL gentamicin throughout the remainder of the experiment to eliminate potentially released bacteria from dead cells into the culture medium during the incubation. Osteoblasts used to evaluate internalization (3 h post-infection) and intracellular persistence (24 post-infection) were lysed via osmotic shock. To quantify the intracellular bacteria, dilutions of cell lysates were plated in duplicate on Trypcase Soy Agar (TSA; bioMérieux, Marcy-L’Etoile, France) plates, followed by incubation overnight at 36°C. To evaluate cytotoxicity, lactate dehydrogenase (LDH) release from damaged cells 24 h post-infection was quantified in the cell culture supernatant based on a colorimetric method using a Dimension Vista automated clinical chemistry analyzer (Siemens Healthcare Diagnostics, Tarrytown, NY, USA).

### DNA Extraction of *S. pseudintermedius* Strains

DNA was extracted after suspending the bacterial colonies in 500 μL of Quick Extract (Epicentre^®^, Madison, WI, USA). A thermal shock was applied; this shock included a first step at 65°C for 30 min and a second step at 98°C for 20 min (PTC 100, Heated Lid Thermal Cycler, MJ Research).

### DNA Amplification of Targeted Genes

Consensus sequences for *spsD* and *spsL* were obtained using Seaview software (version 4.5.4) after aligning the sequences of the genes of interest (*spsD* and *spsL*) obtained from four public whole genome sequence (WGS) genomes of *S. pseudintermedius*: strain ED99 (GenBank acc. num.: CP002478.1), strain K7 (GenBank acc. num.: BARM01000000), strain E140 (GenBank acc. num.: ANOI01000001) and strain HKU10-03 (GenBank acc. num.: CP002439.1). Specific primers targeting the conserved regions in the consensus region of each gene of interest (*spsD* and *spsL*) were designed using Primer 3 software. The reaction mixture used and the PCR program used for amplification are presented in **Supplementary Table S1**.

### Statistical Analysis

Non-parametric statistical Mann–Whitney *U* tests and *B* tests with a threshold of significance of 0.05 were used to determine statistically significant differences. All analyses were performed using XLSTAT software (Pearson Edition, developed by Addinsoft v.2014). The results were expressed as the means and 95% confidence intervals derived from three independent experiments performed in triplicate.

## Results

### Adhesion of *Staphylococcus* Non-*aureus* to Human Fibronectin

**FIGURE 1:**
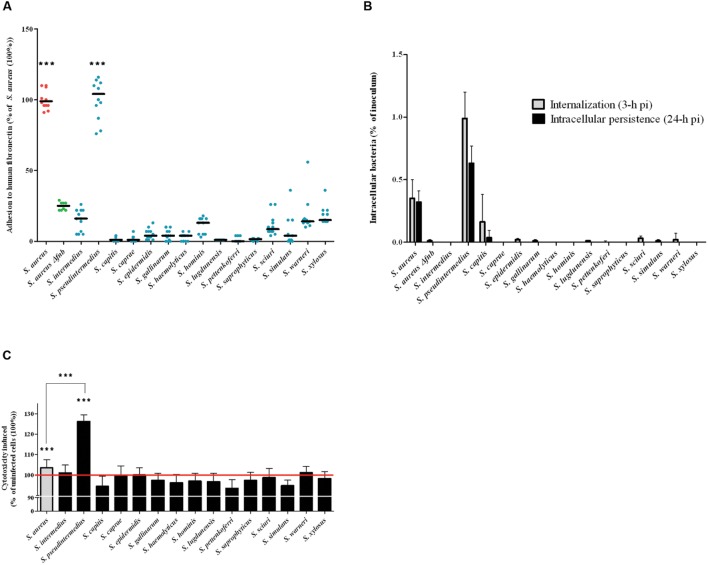
**Evaluation of *Staphylococcus spp.* in terms of their adherence to human fibronectin, their internalization and their persistence in MG63 cells.** **(A)** Quantification of the fibronectin adhesion capacity of 15 *Staphylococcus spp.* reference strains. All of the results are expressed as percentages of the values obtained for the *S. aureus* 8325-4 strain. The horizontal bars denote the means derived from three independent experiments performed in quadruplicate. The fibronectin adhesion capacity of the SNA strains was compared to that of *Δfnb S. aureus* using the one-tailed Mann–Whitney test with an α risk of 0.05. (^∗∗∗^*p* < 0.001; SNA: *Staphylococcus* non-*aureus*). **(B)** MG63 cells were infected for 2 h at 37°C with staphylococci at a multiplicity of infection (MOI) of 100:1 for all strains. The invasion and persistence capacities were assessed by quantifying the viable intracellular bacterial loads at 3 h and 24 h post-infection after gentamicin treatment. Bars represent means ± standard deviations derived from three experiments performed in triplicate, and the results are expressed as the percentages of initial inoculum internalized. **(C)** Quantifications of LDH release, reflecting cytotoxicity, were performed on culture supernatants at 24 h post-infection. All of the results are expressed as the percentages of the values obtained for the control “uninfected cells” (100%), represented by the red line. Bars represent means ± standard deviations derived from three experiments performed in triplicate. The increase in the LDH concentration in the cells infected with an SNA strain compared to the uninfected control cells was evaluated using a one-tailed Mann–Whitney test with an α risk of 0.05 (^∗∗∗^*p* < 0.001; LDH: lactate dehydrogenase; SNA: *Staphylococcus* non-*aureus*; pi: post-infection).

The capacity of each isolate to adhere to fibronectin *in vitro* was evaluated after staining with crystal violet and measuring the OD_620_, which is directly related to the amount of adherent bacteria. *S. aureus* 8325-4 and the DU5883 mutant were used to validate the protocol. The ability of the *fnb*-deleted DU5883 strain to adhere to fibronectin was reduced by 76% compared to the wild type strain (*p* < 0.001, **Figure 1A**). The results, which are expressed as percentages of the *S. aureus* 8325-4 adhesion rate (100%), showed that the adhesion rates of all of the tested SNA isolates were below the adhesion rate the negative control DU5883 (24.87 ± 2.90%). The only exception was *S. pseudintermedius*, which exhibited a significantly higher adhesion rate (100.25 ± 13.72%) than DU5883 (*p* < 0.001). Additionally, the adhesion rate of *S. pseudintermedius* was in the range of the adhesion rates observed for *S. aureus* 8325-4.

### Internalization and Persistence of SNA Isolates in Human Osteoblasts

The internalization (3 h post-infection) and persistence (24 h post-infection) abilities of the strains in MG63 osteoblasts were assessed using a gentamicin protection assay, and the results are expressed as percentages of internalized inoculum (**Figure 1B**). With the exception of *S. pseudintermedius*, all of the tested SNA strains exhibited lower internalization and persistence rates than DU5883 *S. aureus Δfnb*, which was used as the negative control (0.01 ± 0.01% and <0.01%, respectively). In contrast, *S. pseudintermedius* demonstrated significantly higher internalization ability (0.99 ± 0.20%) and persistence (0.63 ± 0.14%) in osteoblasts than *S. aureus* 8325-4 (0.35 ± 0.15% and 0.32 ± 0.09%, respectively; *p* < 0.001). In addition, although *S. capitis* displays internalization ability in osteoblasts, this species cannot persist in the intracellular compartment.

### Cytotoxicity of SNA Isolates to Human Osteoblasts

The cytotoxicity of SNA isolates to MG63 osteoblasts at 24-h post-infection was determined by measuring the levels of LDH released from damaged osteoblasts in the culture supernatant. The cells infected with the reference strain *S. pseudintermedius* exhibited a significantly higher rate of cytotoxicity (126.22 ± 3.27%, *p* < 0.001) than the uninfected cells (100%). This level of cytotoxicity was even higher than the cytotoxicity observed after infection with *S. aureus* 8325-4 (103.52 ± 3.97%, *p* < 0.001). Notably, cells infected with all other SNA species showed no cytotoxicity compared to uninfected cells (100 ± 1.87%; *p* = 0.55, **Figure 1C**).

### Behavior of a Set of Clinical Isolates of *S. pseudintermedius*

**FIGURE 2:**
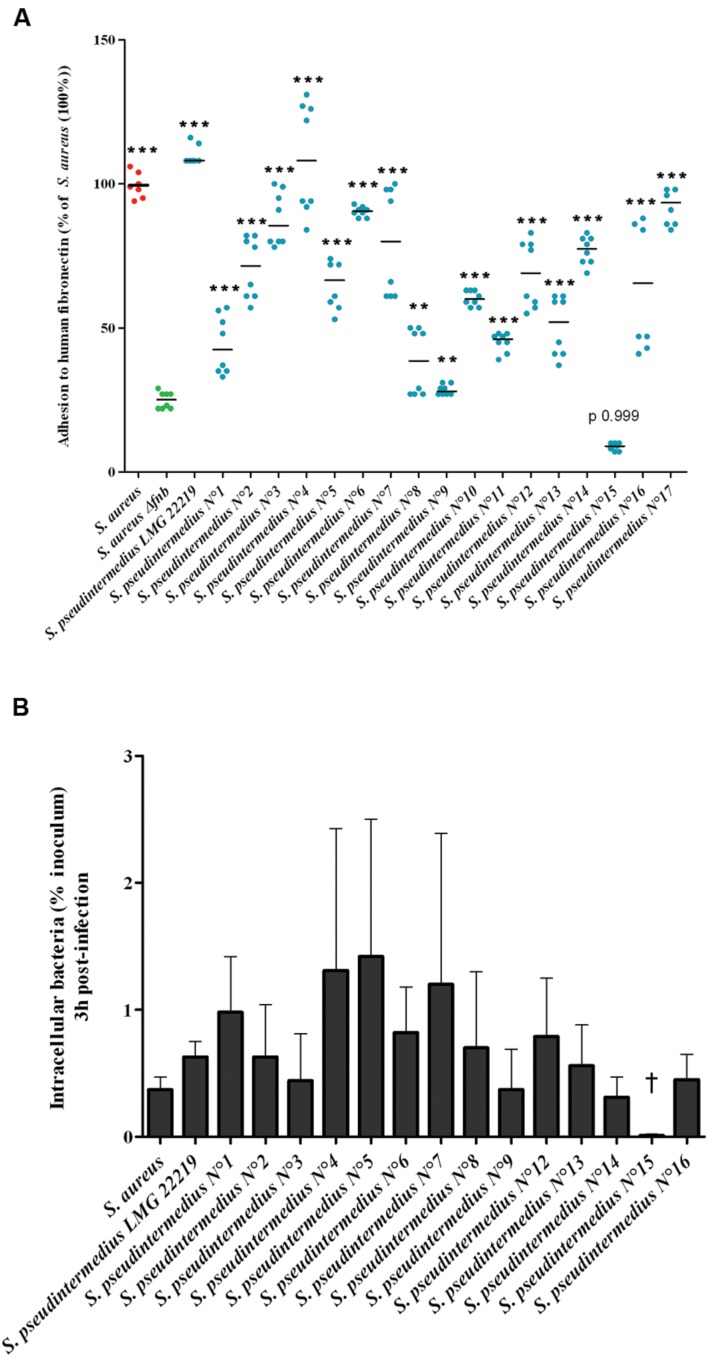
**Fibronectin adhesion and internalization of 17 *S. pseudintermedius* clinical isolates into MG63 osteoblasts.** **(A)** Adhesion of the isolates to human fibronectin was evaluated by measuring the absorbance at 620 nm normalized to the reference strain *S. aureus.* Plots and means were derived from three experiments performed in triplicate. The difference in the capacity of the SNA strains to adhere to fibronectin compared to DU5883 *Δfnb S. aureus* was assessed using the Mann–Whitney one-tailed test with an α risk of 0.05 (^∗∗^*p* < 0.01, ^∗∗∗^*p* < 0.001; OD: optical density). **(B)** Capacity of internalization of *S. pseudintermedius* clinical isolates into MG63 osteoblasts. Bars represent means ± standard deviation derived from two experiments performed in triplicate, and the results are expressed as percentages relative to the initial inoculum († = 0.01 ± 0.01%).

The original results observed for the reference strain *S. pseudintermedius* led us to investigate whether it’s particularly high abilities to adhere to fibronectin and internalize into osteoblasts was species-specific and consequently observed for other *S. pseudintermedius* isolates. Sixteen out of the 17 tested clinical *S. pseudintermedius* isolates adhered significantly more frequently than DU5883 *Δfnb S. aureus* (24.87 ± 2.90%; *p* < 0.01). The adhesion rate of *S. pseudintermedius* isolates varied from 28.54 ± 1.7% to 109.74 ± 2.28% compared to *S. aureus* 8325-4, which was used as a reference (100%; **Figure 2A**). In addition, 14 out of the 15 gentamicin-susceptible isolates exhibited higher internalization and persistence rates than DU5883 *Δfnb S. aureus* based on the gentamicin protection assay (*p* < 0.01 – **Figure 2B** and **Supplementary Figure S1**). Concerning infection-induced cytotoxicity, 14 out of 15 isolates harbored a comparable phenotype to the reference strain, and cells infected with these isolates exhibited a significantly higher cytotoxicity rate (*p* < 0.001) than uninfected cells (100%; **Supplementary Figure S1**).

**FIGURE 3:**
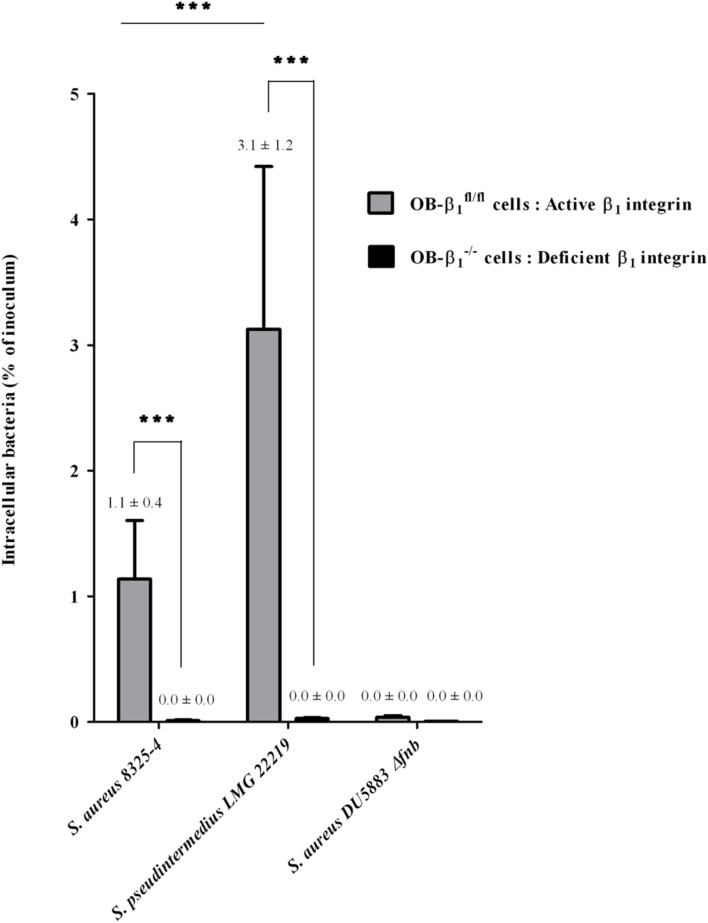
**Determination of the involvement of β1 integrin in the *S. pseudintermedius* internalization process using murine osteoblast cell lines (OB-β_1_^fl/fl^ and OB-β_1_^-/-^) with functional and non-functional β_1_ subunits, respectively.** Internalization capacity of *S. aureus* 8325-4 (positive control), DU5883 *S. aureus Δfnb* (negative control) and the reference strain *S. pseudintermedius* LMG 22219 in OB-β_1_^fl/fl^ and OB-β_1_^-/-^ osteoblasts measured 3 h post-infection derived from three experiments performed in triplicate. The bilateral Mann–Whitney test was used to compare the data obtained for the OB-β_1_^fl/fl^ and OB-β_1_^-/-^ osteoblasts at an α risk of 0.05 (^∗∗∗^*p* < 0.001).

### *spsD* and *spsL* Amplification via PCR

*spsD* and *spsL* (*S. pseudintermedius* surface proteins), the two genes potentially involved in the internalization process due to their high homology to *S. aureus* FnBPs, were investigated. The results of the amplification of both *spsD* and *spsL* showed the presence of these genes in all *S. pseudintermedius* isolates (*n* = 18), including the strain n°15, which was unable to adhere to fibronectin or become internalized.

### Characterization of the Cellular Pathways Involved in the Internalization of *S. pseudintermedius*

To determine if the internalization mechanism of *S. pseudintermedius* in osteoblasts involves the cellular integrin α_5_β_1_, as described for *S. aureus*, an *in vitro* model comparing two murine osteoblast cell lines, OB-β_1_^fl/fl^ and OB-β_1_^-/-^ (i.e., with and without β_1_ integrins, respectively), was used (**Figure 3**). We evaluated the internalization ability of *S. aureus* 8325-4 (positive control), *S. aureus* DU5883 (negative control), and *S. pseudintermedius* LMG 22219 (reference strain). As expected, although the rate of internalization of the OB-β_1_^fl/fl^ cells reached 1.14 ± 0.41% of the inoculum for *S. aureus* 8325-4, a complete loss of internalization was observed for the OB-β_1_^-/-^ cells (0.01 ± 0.01%, *p* < 0.001). Similarly, the *S. pseudintermedius* strain demonstrated a high capacity of internalization among OB-β_1_^fl/fl^ cells (3.12 ± 1.2% of the inoculum), but this capacity was abolished in OB-β_1_^-/-^ cells (0.03 ± 0.02%, *p* < 0.001). Of note, internalization of *S. pseudintermedius* into OB-β_1_^fl/fl^ osteoblasts was 2.7-fold higher than the internalization of the *S. aureus* strain (*p* < 0.001). These data demonstrated that the mechanism underlying *S. pseudintermedius* internalization is β_1_ integrin dependent.

## Discussion

The aim of the present study was to investigate the ability and impact of internalization of various SNA species into osteoblasts, a mechanism implicated in the chronicity of BJIs. A screening approach using 15 reference strains of 15 different SNA species was used to evaluate the capacity of SNA isolates to (i) adhere to fibronectin, (ii) become internalized and persist intracellularly, and (iii) induce the death of human osteoblasts. Our results revealed a homogeneous behavior among the different investigated virulence phenotypes for most of the SNA species tested. Indeed, all of the species except one (14/15) were unable to adhere to fibronectin, to become internalized, to persist in the intracellular compartment and to induce cytotoxicity in human osteoblasts. These observations suggest that internalization into NPPCs is likely not a major pathophysiological mechanism associated with the chronicity and/or recurrence of BJIs with most SNA species.

To our best knowledge, this is the first study to compare the interactions between human NPPCs and a large panel of staphylococcal species under the same experimental conditions. This screening approach, using one strain per species, was used to quickly identify possible atypical features of an SNA species. However, this approach may also represent a limitation because our results may not be generalizable at the species level. Nevertheless, the few studies of SNA species that have included a panel of strains have reported that internalization into NPPCs is a feature shared among SNA species (Valour et al., 2013; Campoccia et al., 2015).

In contrast to the other tested species, *S. pseudintermedius* showed a significant capacity to adhere to fibronectin, to become internalized and to persist in osteoblasts, even more so than *S. aureus*. Using a complementary panel of 17 *S. pseudintermedius* clinical isolates, we confirmed that all of the tested isolates except one (strain n°15) exhibited the same phenotype as the reference strain. Interestingly, the strain that was unable to invade osteoblasts was also the only strain unable to adhere to fibronectin. This observation suggested the involvement of fibronectin in the internalization process and, as a consequence, indicated that fibronectin-binding proteins expressed on the bacterial surface could play a role in the internalization process of *S. pseudintermedius*, as demonstrated in *S. aureus*, via interactions with FnBPA and FnBPB, which are encoded by the *fnbA* and *fnbB* genes, with fibronectin. However, Bannoehr et al. (2011) reported the absence of the *fnbA* and *fnbB* genes from the genomes of *S. pseudintermedius* available in the NCBI database but the presence of two cell wall-anchored (CWA) proteins, SpsD and SpsL, could putatively bind to fibronectin. Concomitantly, in line with our results, a recent study showed that *S. pseudintermedius* is able to be internalized by canine progenitor epidermal keratinocytes (CPEKs), another type of NPPCs (Pietrocola et al., 2015). The authors demonstrated that either SpsD or SpsL is sufficient for the internalization of *S. pseudintermedius* into CPEKs. Here, we detected the presence of these two genes in all of the clinical isolates of this species, including the only isolate that exhibited no adhesion to fibronectin and no internalization. Moreover, using the murine osteoblast cell lines OB-β_1_^fl/fl^ and OB-β_1_^-/-^, we showed that internalization was abolished when the interaction between the bacteria and cellular β_1_ integrin is compromised. This finding confirmed a key role of this integrin in the bacterial internalization process. These results are in agreement with the observations of Pietrocola et al. (2015) who used a different approach based on α_5_β_1_ integrin-blocking antibodies. Taken together, the results concerning the *S. pseudintermedius* cellular invasion process appear to be consistent with those for *S. aureus*, in which 3 effectors are involved in the invasion process*:* (i) the CWA proteins SpsD and SpsL, as opposed to FnBPA and FnBPB in *S. aureus*; (ii) fibronectin; and (iii) the α_5_β_1_ integrins of human and canine NPPCs. This evidence suggests that this mechanism is independent of the cell type and origin.

In addition, after internalization, the intraosteoblastic lifestyle of *S. pseudintermedius* was associated with a high level of cytotoxicity, even greater than that observed for *S. aureus* 8325-4 and a large panel of clinical *S. aureus* isolates (data not shown). This result suggests that *S. pseudintermedius* likely expresses several specific virulence factors in the intracellular compartment. Pietrocola et al. (2015) hypothesized that a leukotoxin similar to the *S. aureus* Panton-Valentine leucocidin (PVL) termed Luk-I, which is encoded by the two co-transcribed genes *lukS* and *lukF*, may be involved in *S. pseudintermedius* virulence (Bannoehr and Guardabassi, 2012; Cheung et al., 2014). In fact, it has been demonstrated that similar to PVL, Luk-I targets human polymorphonuclear leukocytes (Riegel et al., 2011). Moreover, we and others demonstrated that the intracellular cytotoxicity of *S. aureus* is related to phenol-soluble modulin (PSM) expression, although PVL does not contribute to the intracellular cytotoxic activity of the bacteria on osteoblasts (Rasigade et al., 2013). Thus, regarding the invasion process, the mechanism is likely similar for *S. pseudintermedius*, for which cell death is likely involved in the expression of PSMs, whose genes are present in all available *S. pseudintermedius* genomes (Cheung et al., 2014). Notably, the presence of PSMs has been described in the SNA species, although they are unable to be internalized by osteoblasts, as demonstrated in our study. In addition to their involvement in intracellular cytotoxicity, PSMs have been associated with pleiotropic activities (biofilm formation and spreading processes) that could play a role in infections with these species (Tsompanidou et al., 2013; Dastgheyb et al., 2015).

Our results raise interesting questions about *S. pseudintermedius*. In fact, the invasion process of *S. pseudintermedius* mirrors that of *S. aureus*, as cellular invasion involved the SpsD and SpsL proteins for the former and the FnBPA and FnBPB proteins for the latter. The mechanism of NPPCs cytotoxicity also appears to be similar between these species. In fact, even if *S. pseudintermedius* is not a common agent of human disease, it is a major veterinary pathogen responsible for a high proportion of canine infections, including cutaneous infections (such as pyoderma) associated with purulent necrotizing infections (Bannoehr and Guardabassi, 2012) and BJIs (Miedzobrodzki et al., 2010). These severe forms of infection are in agreement and likely for a part related to the high cytotoxicity observed after *S. pseudintermedius* invasion into host cells. The reason underlying the low prevalence of *S. pseudintermedius* in human infections is questionable and is in opposition to the intensive and close contact between humans and pets in which a high prevalence of *S. pseudintermedius* is observed, primarily among dogs [including an increasing prevalence of methicillin-resistant *S. pseudintermedius* (Duim et al., 2015)]. Indeed, only a few sporadic human of *S. pseudintermedius* infection have been reported, mostly after contact with dogs (Van Hoovels et al., 2006; van Duijkeren et al., 2011). These findings can be explained by a specific host tropism, related to the differential ability of *S. pseudintermedius* and *S. aureus* to colonize dogs and humans, respectively. Hanselman et al. (2009) reported rates of *S. pseudintermedius* colonization of 4.1% in humans but of 46% in dogs. This difference could be associated with the presence of (i) specific factors, such as microbial surface components recognizing adhesive matrix molecules (MSCRAMMs), in each of the two staphylococcal species and/or (ii) specific cell-surface targets of MSCRAMMs present on mucosal cells. This hypothesis is reinforced by the findings of a recent study that compared the adhesiveness of human and canine strains of *S. pseudintermedius.* The results showed that the canine strain adhered more efficiently to canine corneocytes than to human corneocytes. Furthermore, strains isolated from humans exhibited similar adhesion abilities between the two cell types; this result suggested an adaption of these strains to colonize a new host (Latronico et al., 2014).

## Conclusion

Screening of a large panel of SNA species showed that persistence in NPPCs is likely not a widespread and predominant pathophysiologic pathway underlying the chronicity of infection with SNA, especially BJI. These findings emphasize the need for studies focused on biofilm formation by SNA species that are implicated in BJIs, which can represent another pathophysiological pathway of BJI persistence. *S. pseudintermedius* appears to be an exception, as this species exhibited abilities to invade and then induce cytotoxicity to NPPCs that were similar to the characteristics of *S. aureus*. This evidence could explain the severe and necrotic forms of infection caused by *S. pseudintermedius* in pets, which exhibit a high prevalence of *S. pseudintermedius* infection.

## Author Contributions

Conceived and designed the experiments: FL and ST-A. Performed the experiments: YM. Data collection: YM. Data analysis: PM, ST-A and YM. Data interpretation: PM, ST-A and FL. Drafting manuscript: YM and ST-A. Revising manuscript content: FV, DB, MB, MH, J-PR, TF, ST-A, and FL. FL takes responsibility for the integrity of the data analysis.

## Conflict of Interest Statement

The authors declare that the research was conducted in the absence of any commercial or financial relationships that could be construed as a potential conflict of interest.
