# Supplementary material for: Pathophysiological Mechanisms of Staphylococcus Non-aureus Bone and Joint Infection: Interspecies Homogeneity and Specific Behavior of S. pseudintermedius
Source: Front Microbiol. 2016 Jul 12;7:1063. doi: 10.3389/fmicb.2016.01063 (PMC4940379; doi:10.3389/fmicb.2016.01063)
Supplement: Supplementary file 1 [file Table_1.PDF]

Supplementary Material

Pathophysiological mechanisms of *Staphylococcus* non-*aureus* bone and joint infection: interspecies homogeneity and specific behaviour of *S. pseudintermedius*

Yousef Maali, Patrícia Martins-Simões, Florent Valour, Daniel Bouvard, Michele Bes, Marisa Haenni, Tristan Ferry, Frederic Laurent [*], Sophie Trouillet-Assant

*** Corresponding author:** Pr. Frédéric Laurent, Centre International de Recherche en Infectiologie, INSERM U1111, CNRS UMR5308, Université de Lyon 1, ENS de Lyon, Team "Pathogenesis of staphylococcal infections", Lyon, France.

Laboratoire de Bactériologie, Groupement Hospitalier Nord, 103 Grande Rue de la Croix-Rousse, 69004 Lyon, France.

Tel: +33 (0) 472 07 18 37 E-mail: frederic.laurent@univ-lyon1.fr

Supplementary Materials

Table 1: Conditions of amplification of the *spsD* and *spsL* genes via PCR.

A: The reaction mixture for the PCR assays. B: Sequences of the primers used for PCR. C: Details of the protocol used for DNA amplification.

A

PCR mixture components	Volume	Concentration/quantity
**Buffer**	2.5 µL	10X
**$MgCl_2$**	0.75 µL	25 mM
**DNTP** (deoxynucleoside triphosphate)	4 µL	20 mM
**Primer 1**	1 µL	10 µM
**Primer 2**	1 µL	10 µM
**Taq polymerase**	0.125 µL	5 U/µL
**DNA**	2 µL	-
**H_2O**	qs 25 µL	-

B

	Primer 1 forward	Primer 2 reverse	Expected size (bp)
spsD	GCAACGCAAGCACAAGAAGA	TAGCCATGATTCTCGGTGCG	574
spsL	TGTGAGCGGTCAGTACGATG	CGGGAAGAAACCAGCATCGA	577

C

PCR Step	Times and temperatures	
**Initial denaturation**	5 min - 94°C	
**Cyclic denaturation**	30 sec - 94°C	
**Cyclic hybridization**	30 sec - 60°C	x25 cycles
**Cyclic elongation**	1 min - 72°C	
**Final elongation**	5 min - 72°C	
