# Supplementary material for: Pathophysiological Mechanisms of Staphylococcus Non-aureus Bone and Joint Infection: Interspecies Homogeneity and Specific Behavior of S. pseudintermedius
Source: Front Microbiol. 2016 Jul 12;7:1063. doi: 10.3389/fmicb.2016.01063 (PMC4940379; doi:10.3389/fmicb.2016.01063)
Supplement: Supplementary file 2 [file Presentation_1.PDF]

Supplementary Material

Pathophysiological mechanisms of *Staphylococcus* non-*aureus* bone and joint infection: interspecies homogeneity and specific behaviour of *S. pseudintermedius*

Yousef Maali, Patrícia Martins-Simões, Florent Valour, Daniel Bouvard, Michele Bes, Marisa Haenni, Tristan Ferry, Frederic Laurent *, Sophie Trouillet-Assant

*** Corresponding author:** Pr. Frédéric Laurent, Centre International de Recherche en Infectiologie, INSERM U1111, CNRS UMR5308, Université de Lyon 1, ENS de Lyon, Team ”Pathogenesis of staphylococcal infections”, Lyon, France.

Laboratoire de Bactériologie, Groupement Hospitalier Nord, 103 Grande Rue de la Croix-Rousse, 69004 Lyon, France.

Tel: +33 (0) 472 07 18 37 E-mail: frederic.laurent@univ-lyon1.fr

Supplementary Figure

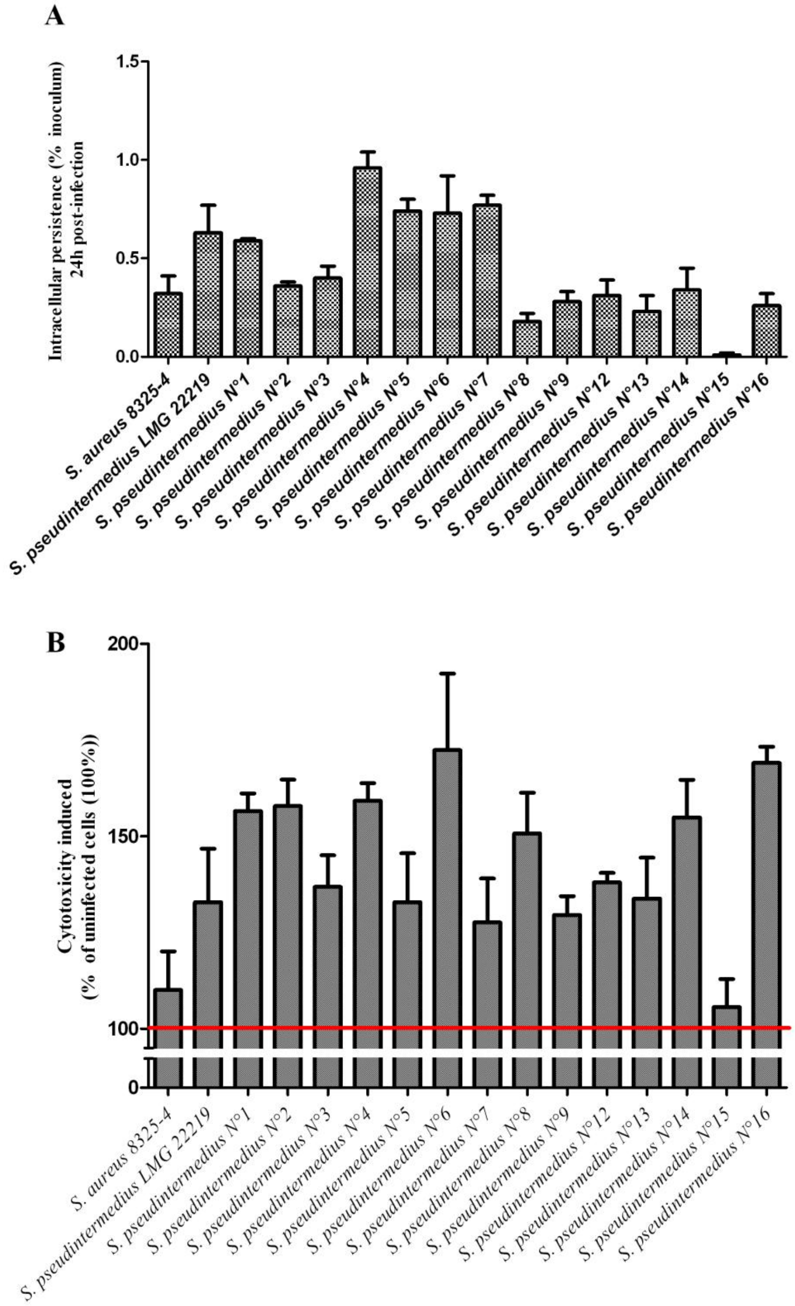

Supplementary Figure 1. Evaluation of the persistence of *Staphylococcus pseudintermedius* isolates in MG63 cells and their ability to induce cytotoxicity. (A) The persistence capacities were assessed by quantifying the viable intracellular bacterial loads at 24 h post-infection after gentamicin treatment. Bars represent means ± standard deviation derived from one experiment performed in triplicate, and the results are expressed as the percentages of the initial inoculum internalized. (B) Quantification of LDH release, reflecting cytotoxicity, was performed on the culture supernatants at 24 h post-infection. All results are expressed as the percentages of the value obtained for the control “uninfected cells” (100%), represented by the red line. Bars represent means ± standard deviation derived from one experiment performed in triplicate.
